# A Benchmark Evaluation of the isoAdvection Interface Description Method for Thermally–Driven Phase Change Simulation

**DOI:** 10.3390/nano12101665

**Published:** 2022-05-13

**Authors:** Ali Yahyaee, Amir Sajjad Bahman, Henrik Sørensen

**Affiliations:** Department of Energy, Aalborg University, 9220 Aalborg, Denmark; hs@energy.aau.dk

**Keywords:** thermally–driven phase change, nanofluids, spurious currents, curvature calculation, volume of fluid, isoAdvection, benchmark cases

## Abstract

A benchmark study is conducted using isoAdvection as the interface description method. In different studies for the simulation of the thermal phase change of nanofluids, the Volume of Fluid (VOF) method is a contemporary standard to locate the interface position. One of the main drawbacks of VOF is the smearing of the interface, leading to the generation of spurious flows. To solve this problem, the VOF method can be supplemented with a recently introduced geometric method called isoAdvection. We study four benchmark cases that show how isoAdvection affects the simulation results and expose its relative strengths and weaknesses in different scenarios. Comparisons are made with VOF employing the Multidimensional Universal Limiter for Explicit Solution (MULES) limiter and analytical data and experimental correlations. The impact of nanoparticles on the base fluid are considered using empirical equations from the literature. The benchmark cases are 1D and 2D boiling and condensation problems. Their results show that isoAdvection (with isoAlpha reconstruct scheme) delivers a faster solution than MULES while maintaining nearly the same accuracy and convergence rate in the majority of thermal phase change scenarios.

## 1. Introduction

Thermally–driven phase change processes are often encountered in various scientific or industrial configurations, including aerospace, power plants, food and other chemical or petrochemical processes. There has been a resurgence of interest and study in the use of thermal phase change processes in conjunction with nanofluids, notably in heat transfer applications involving the flow boiling of nanofluids in recent years [1,2]. Flow boiling is an effective cooling method because the formation of bubbles may remove a significant amount of energy while simultaneously driving the flow due to the density differential. Additionally, the concept of utilizing metallic nanoparticles to enhance the thermal characteristics of liquids was pioneered by Choi and Eastman [3] and has since been the topic of several experimental [4,5,6,7,8] and numerical [9,10,11,12,13,14] studies. Thus, devices that use flow boiling of nanofluids must be capable of absorbing a large amount of heat while also maintaining a uniform temperature distribution across the device.

During the phase-change phenomena, in-depth knowledge about flow and heat transfer mechanisms is required to improve system performance and avoid accidental off-design situations in the applications above. This highlights the importance of studies within thermally–driven phase-change processes. Studies in this area are primarily limited to experiments, but the high pressures typically involved in such experiments commonly hinder to which extent the problem can be investigated. In another problematic case, large heat fluxes are required in micro-scale condition applications, restricting the construction of test setups for experiments. This deficiency of experimental data makes developing accurate, physically consistent, and cost-effective numerical methods essential for thermally–driven phase change applications. Moreover, a significant advantage of numerical modeling of thermally phase change problems is in achieving accurate and complete data of flow and temperature fields in all operating conditions [15,16,17].

However, continuous deformation of the liquid–vapor interface, small spatial–temporal scale, contact line movement, and the high non-linearity of governing equations impose significant complexities on numerical solutions of thermal phase change problems [18]. One of the biggest obstacles in simulating such flows is locating the interface position between two phases. Locating the interface locations is quite problematic due to the presence of severe property gradients across the interface, which expands and undergoes significant deformations and topological changes [19,20]. Different two-phase modeling interface description methods are classified into interface capturing (volume methods) and interface tracking (interface methods). These approaches and their classes will be described shortly further.

The first presented approach is called interface tracking. The main predicament with the interface-tracking approach is that surfaces may appear, merge or disappear in an arbitrarily large interface deformation. This is particularly problematic as the volumes break apart or coalesce such as the complex phenomena of bubble breaking.

On the contrary of the interface-tracking approach, the interface-capturing approach can simulate problems in which interfaces undergo topology changes such as bubble merging and collapsing [21]. This approach consists of an implicit representation of the phases in each cell with an additional scalar field. The three most well-known classes of the interface capturing approach are VOF [22], level set [23] and phase-field [24].

In this research, OpenFOAM (version 2006) [25], which is a C++ open-source CFD library, is used. Among different classes of interface-capturing approach, VOF can be considered as the default implemented one in OpenFOAM. It is also used in numerous studies to simulate flow boiling of nanofluids and thermally–driven phase change phenomena [26,27,28,29,30,31,32]. Abedini et al. [26] investigated the subcooled boiling of alumina-water nanofluid in both a vertical concentric annulus and vertical tube using the VOF technique and mixture model, demonstrating that this approach is capable of accurately predicting the temperature distribution and axial vapor volume percentage. Variations in the vapor volume fraction under constant velocity and mass flux circumstances at the intake are explored and compared over a range of nanoparticle concentrations. Soleimani et al. [28] used the VOF model to simulate extremely subcooled flow boiling of HFE-7100 with varying concentrations of alumina nanoparticles in a microchannel heat sink. Zhang et al. [27] used the VOF technique to analyze numerically the heat transfer and pressure drop characteristics of gas–liquid Taylor flows in a small tube. The liquid was a CuO/water nanofluid, and the gas was nitrogen. Their findings show that the primary cause for heat transfer enhancement and pressure drop penalty is an increase in the thermal conductivity and viscosity of nanofluids.

The VOF method, which is primarily developed by Hirt [22], has become a standard in commercial and open-source CFD software. In the VOF method, a volume fraction field (α) is used to separate the fluids in the domain. The interface is located in the cells where α lies between 0 and 1 where α=1 corresponds to the cell being filled with fluid 1 and α=0 corresponds to the cell cell being filled with fluid 2. The fluids deal with a single set of momentum equations in this model, and the volume fraction of each phase in each cell is tracked throughout the domain.

Various approaches have been utilized to simulate phase change issues in VOF in order to acquire an accurate curvature and to smooth the discontinuous physical characteristics of the interfaces. VOF can be classified into the algebraic VOF and the geometric VOF.

The algebraic VOF solves the volume fraction transport equation using a high-resolution scheme with compressive differencing; no geometric operations are used. Commercial software packages use improved variants of the algebraic VOF approach, e.g., Compressive Interface Capturing Scheme for Arbitrary Meshes (CICSAM) [33] in ANSYS Fluent and High-Resolution Interface Capturing (HRIC) [34] in STAR-CCM+, as well as the MULES included in the open-source computational fluid dynamics software OpenFOAM.

MULES is the default interface description method in OpenFOAM phase change solvers and has been used in extensive phase change problems. The VOF class has a non-sharp character, meaning it produces a smeared interface between phases, resulting in the non-accurate calculation of interfacial properties and generating spurious currents. The existence of non-physical spurious currents leads to the increased interfacial mass transfer while simulating condensation and evaporation [35,36,37,38,39]. The mentioned scenario contributes to high numerical errors in such simulations and can be encountered as the chief shortcoming of VOF.

While simulating phase change by MULES, in order to avoid spurious currents, some complementary methods, called geometric VOF, may be utilized. These geometric volume fraction approaches need more geometric computations to update the volume fraction, resulting in a considerably more refined interface than the algebraic method. The most well-known implementations of the geometric VOF methods include the SOLA-VOF [22], Simple Line Interface Calculation (SLIC) [40], Piecewise Linear Interface Calculation (PLIC) [41] and isoAdvection [42].

Among the aforementioned geometric VOF methods, the isoAdvection method is showing promising results [43] and is also implemented in OpenFOAM. The isoAdvection is a geometric VOF method; it can work on both structured and unstructured meshes; and there are no requirements for cell shapes. Different studies have been done by the isoAdvection method [42,43,44,45,46,47], showing that this method reduce the spurious flows close to the interface.

To the best of the authors’ knowledge, both default thermally–driven phase change solvers in OpenFOAM and earlier research on the flow boiling of nanofluids use VOF-MULES to capture the interface, which results in smeared interfaces, spurious flows and erroneous results. Series of studies in the literature have identified VOF-isoAdvection as an interface description method that can help avoid smeared interfaces. Therefore, it is used for the first time in this paper and for thermal phase change simulations of nanofluids by incorporating a new solver in OpenFOAM. Its simulation results are compared to those of VOF using benchmark cases to highlight their relative strengths and weaknesses in various phase change scenarios. Benchmark cases include

- Stefan problem;
- Horizontal film condensation;
- Film condensation on a vertical wall; and
- 2D film boiling.

These benchmark cases are 1D boiling, 1D condensation, 2D condensation, and 2D boiling, respectively, and they were selected due to their relevance in various processes.

## 2. Numerical Formulation

### 2.1. Flow Governing Equations

The conservation of mass, momentum, energy, and interface description advection equations [42] for two incompressible and immersible fluids are expressed, respectively, as

(1) $$\frac{\partial \rho }{\partial t}+\nabla \cdot \left(\rho U\right)=0,$$

(2) $$\frac{\partial (\rho U)}{\partial t}+\nabla \cdot \left(\rho UU\right)=-\nabla p+\nabla \cdot \tau +\rho g+F,$$

(3) $$\frac{\partial (\rho c_{p}T)}{\partial t}+\nabla \cdot \left(\rho c_{p}UT\right)=\nabla \cdot \left(k\nabla T\right)-m{}^{\prime \prime \prime }\left(h_{v}-h_{l}\right),$$

(4) $$\frac{\partial \alpha }{\partial t}+U\cdot \nabla \alpha =-m{}^{\prime \prime }\left[\frac{1}{\rho_{l}}-\alpha \left(\frac{1}{\rho_{l}}-\frac{1}{\rho_{v}}\right)\right],$$

where $m{}^{\prime \prime \prime }$ is the transferred volumetric mass flux rate and calculated using the Tanasawa model [48]. The interface mass flux is computed in this model as a function of the local interface superheat temperature ($\Delta T_{\mathrm{sat}}$) as

(5) $$m{}^{\prime \prime \prime }=\frac{2\gamma }{2-\gamma }{\left(\frac{M_{l}}{2\pi R}\right)}^{1/2}\frac{\rho_{v}\left(h_{v}-h_{l}\right)\Delta T_{\mathrm{sat}}\left|\nabla \alpha \right|}{T_{\mathrm{sat}}^{3/2}}$$

where γ is the evaporation coefficient (which is adjusted to 1 in this case based on early test benchmarks), $M_{l}$ is the fluid’s molecular weight, and *R* is the universal gas constant.

The fluid domain of a single mixture is specified, with the function α used to distinguish the two fluids. Volumetric surface tension forces and fluid physical properties are calculated differently depending on the technique employed to define the interface. The interface description is given in this article utilizing two distinct interface capturing methods: one is based on an algebraic VOF (MULES), and the other is based on a geometric VOF (isoAdvection). The VOF method employs volume fraction function (α) to represent the interface and individual phases,

(6) $$\alpha =\frac{V_{l}}{V}=\left\{\begin{array}{cc} 1 & \mathrm{nanofluid}\;\left(\mathrm{liquid}\right)\;\\ 0<\alpha <1 & \mathrm{Interface}\;\\ 0 & \mathrm{Vapor}\; \end{array}\right.,$$

where α represents the volume fraction of nanofluid (or in general liquid), $V_{l}$ shows the nanofluid volume within a cell, and *V* is the whole volume of the cell. As shown in Equation (6), α=1 and α=0 represent the points with 100% of liquid and 100% of vapor, respectively, while at the interface of the two phases, we have 0<α<1. The nanofluid volume fraction function (α) is also used to calculate the mixture density ($\rho_{m}$), mixture viscosity ($\mu_{m}$), mixture constant pressure specific heat ($c_{p,m}$), and thermal conductivity ($k_{m}$).

(7) $$\rho_{m}=\alpha \rho_{l}+\left(1-\alpha \right)\rho_{v},$$

(8) $$\mu_{m}=\alpha \mu_{l}+\left(1-\alpha \right)\mu_{v},$$

(9) $$c_{p,m}=\alpha c_{p,l}+\left(1-\alpha \right)c_{p,v},$$

(10) $$k_{m}=\alpha k_{l}+\left(1-\alpha \right)k_{v}.$$

The VOF method adds an additional divergence term to the α advection equation to preserve the conservativeness, convergence, and boundedness [49], contributing only to the interface region and will be zero in liquid or vapor phases. The advection equation is reformulated as Equation (11). This modified volume fraction advection equation is solved utilizing MULES. The isoAdvection solver is similar to the MULES solver except for the interface advection stage. Both of them solve the governing system of equations separated by pressure–velocity coupling using the PIMPLE method.

(11) $$\frac{\partial \alpha }{\partial t}+U\cdot \nabla \alpha +\nabla \cdot \left(\alpha \left(1-\alpha \right)U_{c}\right)=-m{}^{\prime \prime }\left[\frac{1}{\rho_{l}}-\alpha \left(\frac{1}{\rho_{l}}-\frac{1}{\rho_{v}}\right)\right],$$

where $U_{c}$ denotes the compressive velocity, which is determined in the normal direction to the contact in order to prevent dispersion, as

(12) $$U_{c}=\min\left(C_{\alpha }\left|U|,\max|U\right|\right)\frac{\nabla \alpha }{\left|\nabla \alpha \right|},$$

where $C_{\alpha }$ is a compressive factor utilized to improve compression, and it constrains the interface smearing. This value is typically set in the range of unity ($1.0<C_{\alpha }<4.0$) [50].

The isoAdvection can be used as a replacement of the MULES to compress the interface. The isoAdvection method uses the concept of isosurfaces to calculate more accurate face fluxes for interface cells [42]. In each time step, an explicit isosurface is reconstructed for each interface cell. This newly reconstructed isosurface prevents the creation of the diffusive interface representation as to the VOF solvers. While using this isosurface, the movement of the face–interface intersection line (line created as the fluid interface plane in a cell intersects the cell face) is modeled in a time step to obtain an accurate estimate for the volume of fluid transported across each face. For more detailed information on implementation and governing equations in the isoAdvection method, the reader is referred to the article by [42].

### 2.2. Nanofluid Governing Equations for Implementation

Nanofluid phase change phenomena is studied in this work. Nanofluids can be considered homogenous, and their corresponding characteristics can be determined empirically or theoretically. The following equations [51,52,53,54] are used to calculate the density, viscosity, thermal conductivity, specific heat, and surface tension of the nanofluid:

(13) $$\rho_{\mathrm{nf}}=\phi_{\mathrm{np}}\rho_{\mathrm{np}}+\left(1-\phi_{\mathrm{np}}\right)\rho_{\mathrm{bf}},$$

(14) $$\mu_{\mathrm{nf}}=\left(1+2.5\phi_{\mathrm{np}}\right)\mu_{\mathrm{bf}},$$

(15) $$c_{p,\mathrm{nf}}=\frac{\phi_{\mathrm{np}}\rho_{\mathrm{np}}c_{p,\mathrm{np}}+\left(1-\phi_{\mathrm{np}}\right)\rho_{\mathrm{bf}}c_{p,\mathrm{bf}}}{\rho_{\mathrm{nf}}},$$

(16) $$k_{\mathrm{nf}}=\frac{k_{\mathrm{np}}+\left(n-1\right)k_{\mathrm{bf}}-\left(n-1\right)\phi_{\mathrm{np}}\left(k_{\mathrm{bf}}-k_{\mathrm{np}}\right)}{k_{\mathrm{np}}+\left(n-1\right)k_{\mathrm{bf}}+\phi_{\mathrm{np}}\left(k_{\mathrm{bf}}-k_{\mathrm{np}}\right)},$$

(17) $$\frac{\sigma_{\mathrm{bf}}-\sigma_{\mathrm{nf}}}{\sigma_{\mathrm{bf}}}=b\;\ln\left(\frac{\phi_{\mathrm{np}}}{a}+1\right),$$

where $\phi_{\mathrm{np}}$ represents the nanoparticles’ volumetric concentration. The value *n* denotes the empirical shape factor, which for spherical particles equals three. The subscripts np, bf, and nf denote the characteristics of nanoparticles, base fluid, and nanofluid, accordingly. The experimental factors a and b are $7.673\times 10^{-7}$ and $-7.773\times 10^{-3}$, respectively [54].

### 2.3. Discretization Schemes and Criterion for Solution Algorithm

OpenFOAM offers several built-in numerical schemes for the discretization of each term of each conservation equation. In this study, for all OpenFOAM computations in different cases, second-order schemes were adopted. The temporal term is discretized implicitly with the backward scheme, which is a second-order bounded scheme. The convective terms in energy and momentum equations are discretized with vanLeer and vanLeerV schemes, respectively. The additional convective term added in the momentum equation for the compression velocity is discretized with the interfaceCompression scheme. The viscous and terms in the momentum equation are discretized with Gauss Linear and Gauss Linear corrected schemes, respectively, which are central difference schemes. isoAlpha is used as reconstruction scheme for isoAdvection method.

The pressure terms are solved using the generalized Geometric-Algebraic Multi-Grid (GAMG) linear solver, whereas the velocity terms are solved using the smooth solver. The solution is converged if the residual value for pressure and velocity approaches $10^{8}$, while temperature and phase fraction approach $10^{10}$. The primary solution algorithm is based on a combination of the PISO and SIMPLE procedures, which is called PIMPLE in OpenFOAM. The selection of the PIMPLE algorithm supports under-relaxation factors, enhancing the convergence and robustness of the simulation. The pimple algorithm is used in conjunction with three PISO correctors (with nCorrectors set to three), which results in the pressure field being corrected three times each PISO corrector loop. The PIMPLE method was performed three times every time step (with nOuterCorrectors set to three), implying that the pressure–momentum coupling is calculated three times in a single time step. Setting momentumPredictor to true is required for isoAdvection accuracy. The momentumPredictor is a toggle switch that enables/disables the predictor step in the PISO method. The number of non-orthogonal correctors is set to zero (nNonOrthogonalCorrectors).

## 3. Benchmark Cases

This section compares the MULES and isoAdvection methods results implemented in an OpenFOAM thermally–driven phase change solver. The benchmark cases have been chosen from classic cases. In these cases, vapor and liquid are considered to be incompressible with unchanging characteristics. Considering there is no sharp interface, the isosurface of α=0.5 is considered as the vapor–liquid interface. Their phases are initially in quiescent equilibrium. Analytical solutions and experimental correlations are considered the fundamental base of comparison to quantify the results for different benchmark cases.

The correctness of the solver to study a benchmark case is tested via systematic grid refinement. We uniformly divide rectangular domains in our benchmark cases, with $N_{k}$ being the number of nodes in the domain. For example, we take the values $N_{k}=\left[25,75,125,175\right]$ in the first two cases. If $e_{k}$ represents the error produced by partitioning the domain $N_{k}$ times (with [25,75,125,175]), the convergence rate ($R_{k}$) is defined as

(18) $$R_{k}=\frac{\log(e_{k}/e_{k-1})}{\log(N_{k}/N_{k-1})}.$$

### 3.1. Stefan Problem

**Figure 1:**
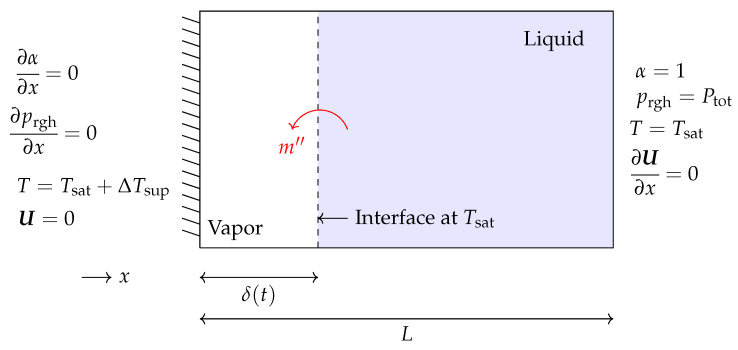
Schematic of the 1D Stefan problem and its corresponding boundary conditions.

The Stefan problem is a benchmark case typically used to validate thermal phase change phenomena in a new solver. Figure 1 presents the schematic of a 1D Stefan problem and its corresponding boundary conditions. As shown, the right boundary is considered to be fixed at saturation temperature ($T_{\mathrm{sat}}=453.03$ K) and is covered by liquid. In contrast, the left boundary is fixed at a superheated temperature with a temperature difference of $\Delta T_{\sup}=10\;K$, and it is covered by vapor. The vapor experiences an increase of temperature at the left wall, which drives mass transfer at the interface and its motion to the right. The thermophysical properties used to solve this test case can be seen in Table 1.

The vapor film thickness (δ) is studied as a function of time. The analytical solution by [55] for the thickness of vapor film is

(19) $$\delta \left(t\right)=2\epsilon \sqrt{\frac{k_{v}t}{\rho_{v}c_{p,v}}},$$

where ϵ is a constant calculated by

(20) $$\epsilon \;\exp\left(\epsilon^{2}\right)\;\mathrm{erf}\left(\epsilon \right)=\frac{c_{p,v}\Delta T_{\sup}}{\left(h_{v}-h_{l}\right)\sqrt{\pi }}.$$

Furthermore, the temperature profile (T(x,t)) along the domain is given as

(21) $$T\left(x,t\right)=T{|}_{x=0}-\frac{\Delta T_{\sup}}{\mathrm{erf}(\epsilon )}\;\mathrm{erf}\left(\frac{x}{2\sqrt{\frac{k_{v}t}{\rho_{v}c_{p,v}}}}\right).$$

The 1D Stefan problem is solved for $N_{k}=\left[25,75,125,175\right]$ number of nodes, and the results are presented as the dimensionless vapor film thickness ($\delta^{*}$) and Jakob number distribution (Ja) for MULES and isoAdvection methods. Vapor film thickness and time are normalized by scaling with the domain length (*L*) and the Capillary time ($t_{\sigma }$), which is calculated by

(22) $$t_{\sigma }=\sqrt{\frac{\rho_{v}L^{3}}{\sigma }}.$$

The Jakob number is created here to offer a dimensionless representation of temperature distribution and is defined by

(23) $$\mathrm{Ja}=\frac{c_{p,v}\left(T-T_{\mathrm{sat}}\right)}{\left(h_{v}-h_{l}\right)}.$$

**Figure 2:**
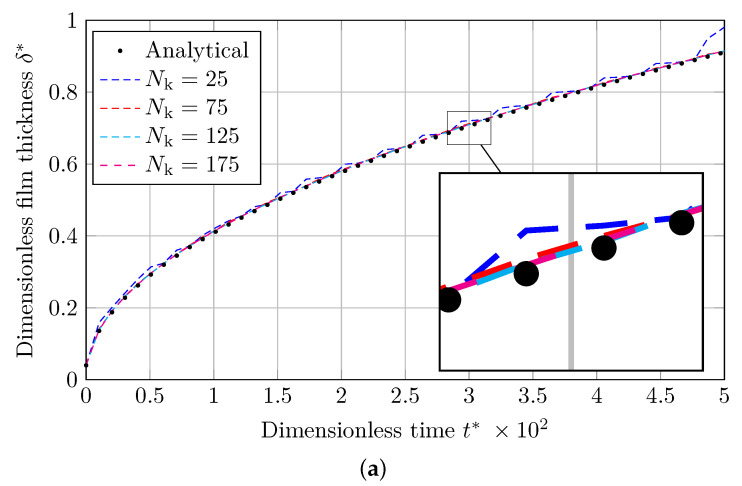

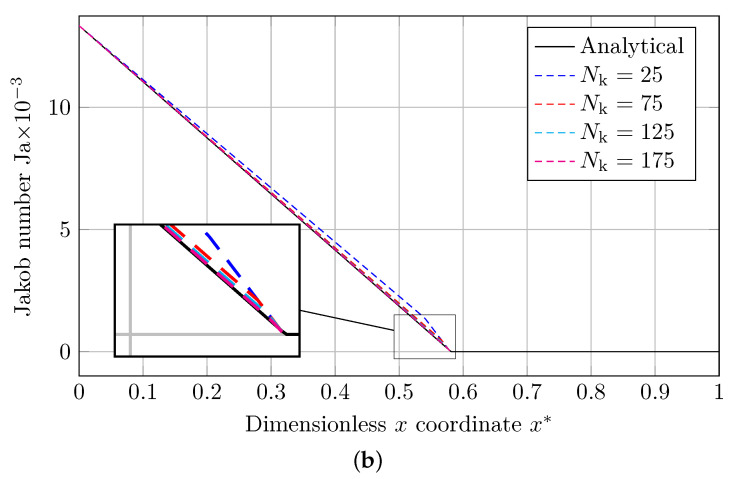
Comparing the analytical solution to the Stefan problem with the numerical results provided by MULES. (**a**) Evolution of dimensionless thickness of the vapor film with dimensionless time. (**b**) Jakob number distribution along the dimensionless domain length.

**Figure 3:**
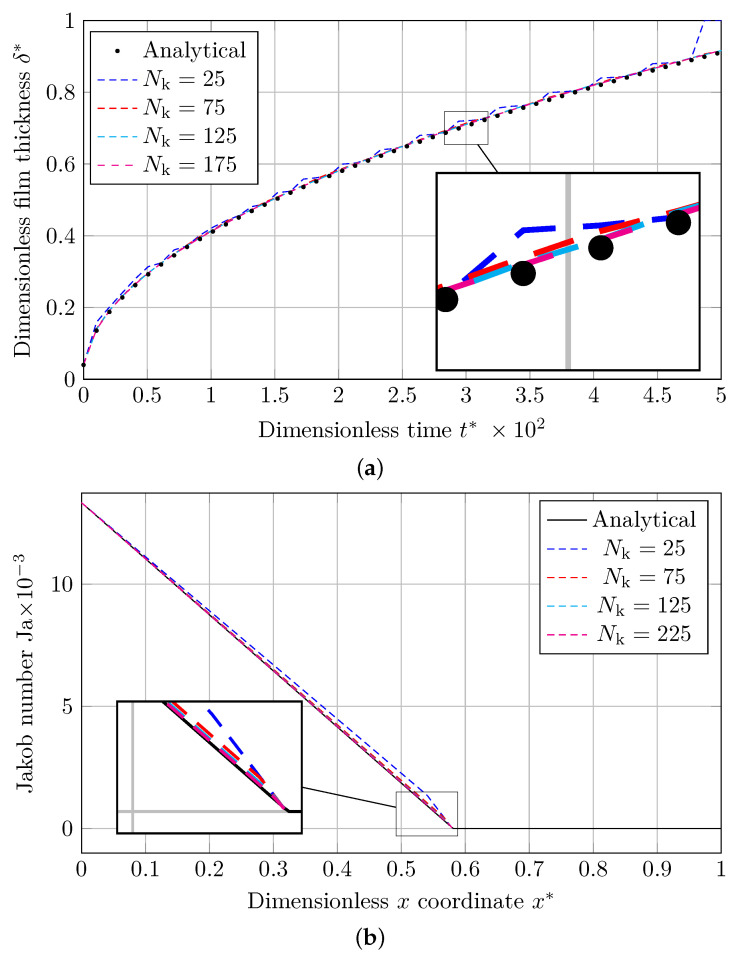
Comparison of the analytical solution to the Stefan problem with the numerical solutions obtained via isoAdvection. (**a**) Evolution of dimensionless thickness of the vapor film with dimensionless time. (**b**) Jakob number distribution along the dimensionless domain length.

As shown in Figure 2a and Figure 3a, graphs for the dimensionless vapor film thickness ($\delta^{*}$), simulated with the coarsest mesh ($N_{k}=25$), are associated with fluctuations. As the grids are refined, these fluctuations are damped, and the result approaches the analytical solution.

The Jakob number along dimensionless *x* coordinate ($x^{*}$) at t=20 s is presented in Figure 2b and Figure 3b. As shown, the Jakob number before and adjacent to the liquid–vapor interface does not entirely agree with the analytical solution, especially at simulations using coarser grids ($N_{k}=\left[25,75\right]$). This is because a source term implicitly forces the interface temperature to be the saturation temperature [56,57].

To define the error between simulation results and the analytical results, we use the definition of the L2 error norm. The following equation is used to determine the L2 norm of the error for the temperature distribution ($e_{T}$) for various grid sizes

(24) $$e_{T}=\sqrt{\frac{\sum_{i=1}^{N_{k}}{\left(\frac{T_{\mathrm{num}},i-T_{\mathrm{ana}},i}{T_{\mathrm{ana}},i}\right)}^{2}}{N_{k}}}.$$

**Figure 4:**
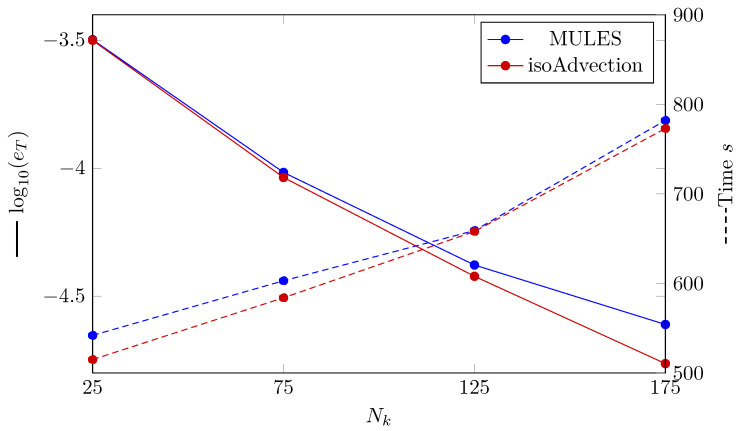
(Solid lines) Logarithmic error presentation, where $e_{T}$ is the L2 norm of error determined by Equation (24). (Dashed lines) Computation time of MULES and isoAdvection techniques to solve the Stefan problem at various grid sizes.

The temperature distribution’s L2 norm of error is presented in Figure 4. The convergence with increasing the number of nodes is observed for both MULES and isoAdvection. It can be seen that the error produced by isoAdvection is lower than MULES. Based on this figure, the convergence rate (Equation (18)) between the two finest grids ($N_{k}=\left[125,175\right]$) for isoAdvection and MULES are 2.33 and 1.58, respectively. In Figure 4, also, the analyzed computational time (presented as dashed lines) for the different number of grids is presented. As shown for all grid sizes, the isoAdvection method provides a faster solution.

### 3.2. Horizontal Film Condensation

**Figure 5:**
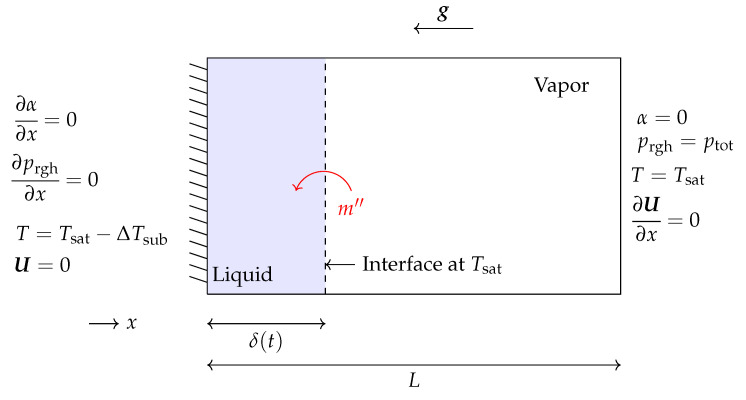
A representation of the 1D horizontal film condensation case and associated boundary conditions.

In this section, we present simulations of horizontal film condensation on an infinite plane and compare the results with Nusselt’s film theory [58]. The schematic of the test case with the applied boundary conditions can be seen in Figure 5. The right wall is the free stream at saturation temperature ($T_{\mathrm{sat}}=453.03$ K), and the left wall is held at a subcooled temperature with $\Delta T_{\mathrm{sub}}=30$ K. On the left wall, the vapor condenses to create a liquid film. A control volume analysis gives an analytical approach for determining the film thickness ($\delta_{\mathrm{an}}\left(t\right)$) as a function of time assuming a linear temperature distribution from sub-saturated to saturation, as

(25) $$\delta_{\mathrm{an}}\left(t\right)={\left[2t\left(\frac{k_{l}}{\rho_{l}c_{p,l}}\right){\left(\frac{1}{2}+\frac{h_{v}-h_{l}}{c_{p,l}\Delta T_{\mathrm{sub}}}\right)}^{-1}\right]}^{\frac{1}{2}}.$$

The thermo-physical properties utilized in this test case can be seen in Table 1. A four-mesh structure with $N_{k}=\left[25,75,125,175\right]$ number of grids in the *x* direction are chosen to study. The case is initialized with the interface location at δ(0)/L=0.015.

**Figure 6:**
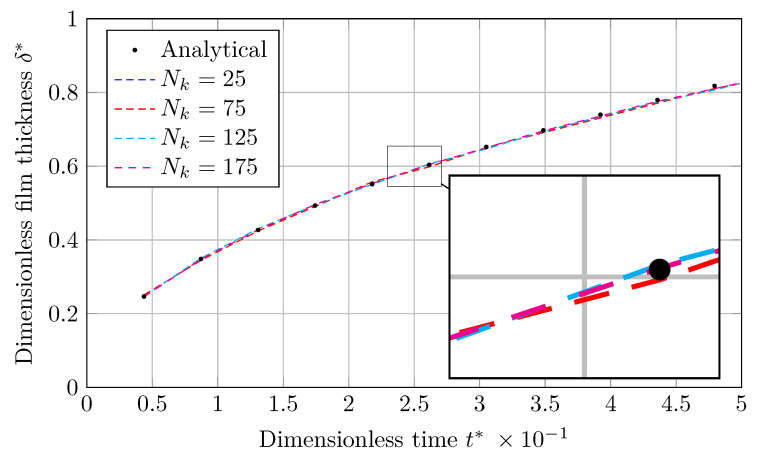
Evolution of dimensionless thickness of condensed liquid film with dimensionless time obtained by MULES in horizontal film condensation case.

**Figure 7:**
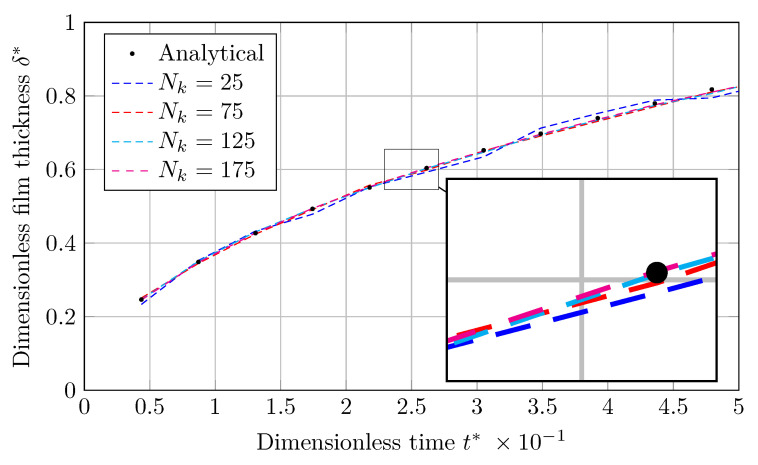
The development of dimensionless thickness of a condensed liquid film with dimensionless time produced via isoAdvection in the horizontal film condensation case.

The output will be in the form of liquid film thickness (δ) against time (*t*), which will be dimensionless by scaling with *L* and $t_{\sigma }$ (Equation (22)), respectively. Figure 6 and Figure 7 show the evolution of dimensionless condensed liquid film thickness ($\delta^{*}$) vs. dimensionless time ($t^{*}$) using the MULES and isoAdvection techniques, respectively. The findings match the analytical results for all mesh sizes, as displayed.

**Figure 8:**
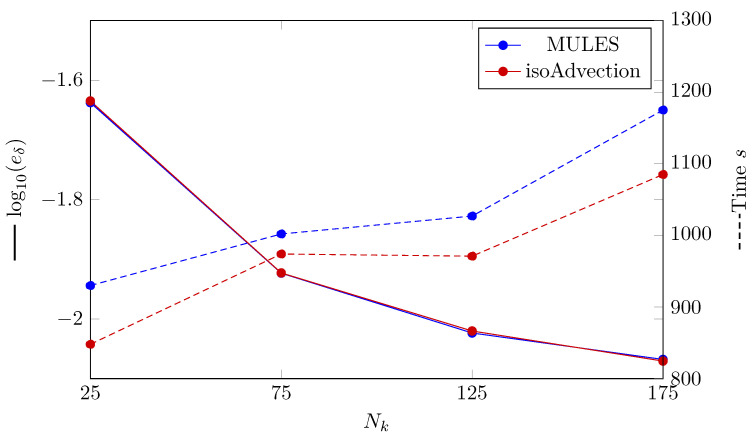
(Solid lines) Logarithmic error presentation, where $e_{\delta }$ is the L2 norm of error determined by Equation (26). The computation time of MULES and isoAdvection algorithms at various grid sizes to solve the horizontal film condensation problem (dashed lines). The number of nodes along the *x* axis is shown on the horizontal axis ($N_{k}$).

In a similar vein to the Stefan problem, the convergence of the MULES and isoAdvection methods in the horizontal film condensation case is compared by calculating the L2 error (Figure 8). The L2 error for condensed liquid film thickness is defined as

(26) $$e_{\delta }=\sqrt{\frac{\sum_{i=1}^{N_{\delta t}}{\left(\frac{\delta_{\mathrm{num}},i-\delta_{\mathrm{ana}},i}{\delta_{\mathrm{ana}},i}\right)}^{2}}{N_{\delta t}}},$$

where $N_{\delta t}$ is the number of time steps. As it can be seen in Figure 8, MULES and isoAdvection deliver results with the same accuracy. The convergence rate (Equation (18)) between the finest grid structures ($N_{k}=\left[125,175\right]$) for both methods is similar to each other and equal to 0.32. The computational time study may also be viewed in Figure 8. As demonstrated in the graph, isoAdvection outperforms MULES in terms of computing speed while maintaining the same accuracy.

### 3.3. 2D Laminar Film Condensation on a Vertical Plate

**Figure 9:**
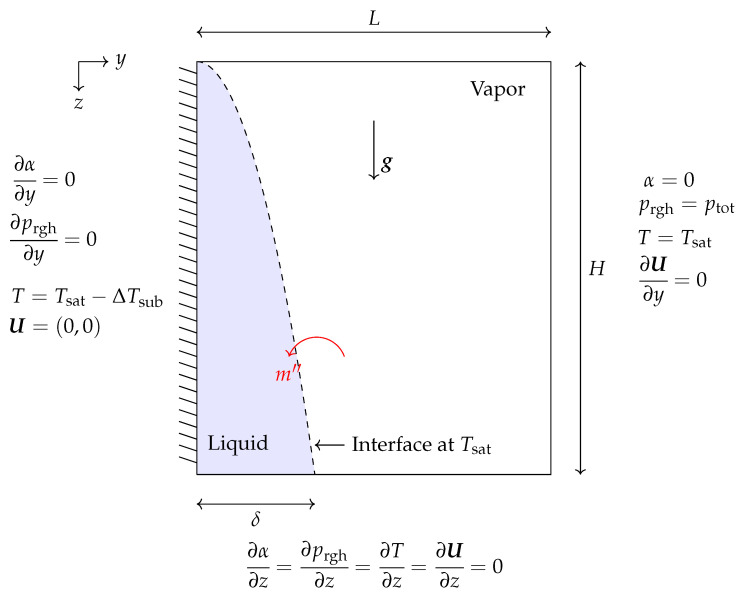
The schematic showing the geometry and boundary conditions for laminar film condensation on a vertical wall case.

In this verification test, a vertical wall is used to simulate film condensation. The simulation domain has dimensions of L×H in the *y* and *z* axes, respectively. The simulations began with a stationary liquid film of uniform thickness $\delta_{0}$ on the left wall. Four grid structures of $N_{k}=\left[25\times 50,75\times 150,125\times 250,175\times 350\right]$ are chosen to study this benchmark case. The grid distribution is graded by the factor of 10 in *y* and *z* directions to produce refined mesh cells in the area of the liquid film. As shown schematically in Figure 9, the hydrophilic flat plate is suspended in a large volume of vapor at the saturation temperature ($T_{\mathrm{sat}}=646$ K). The plate is kept at a constant, subcooled temperature with $\Delta T_{\mathrm{sub}}=20$ K. Zero gradient boundary conditions were applied for the top and bottom of the domain for α, *T*, U, and $p_{\mathrm{rgh}}$. The analytical solution is provided in the literature [58,59], assuming a linear temperature distribution throughout the condensate and disregarding interface shearing stress and inertia forces. Since the gravity forces are working in the *z* direction, the condensed film thickness will grow when *z* increases. The analytical solution for the film thickness (δ) as a function of *z* coordinate is given as

(27) $$\delta ={\left[\frac{4\mu_{l}k_{l}\Delta T_{\mathrm{sub}}z}{g\left(h_{v}-h_{l}\right)\rho_{l}\left(\rho_{l}-\rho_{v}\right)}\right]}^{\frac{1}{4}}.$$

The material properties are shown in Table 2. The liquid film thickness (δ) and *z* coordinate are normalized by scaling with *L*, and the *y* coordinate is normalized by scaling with *H*. The geometric parameters values for the laminar film condensation on a vertical wall case can be seen in Table 3.

**Figure 10:**
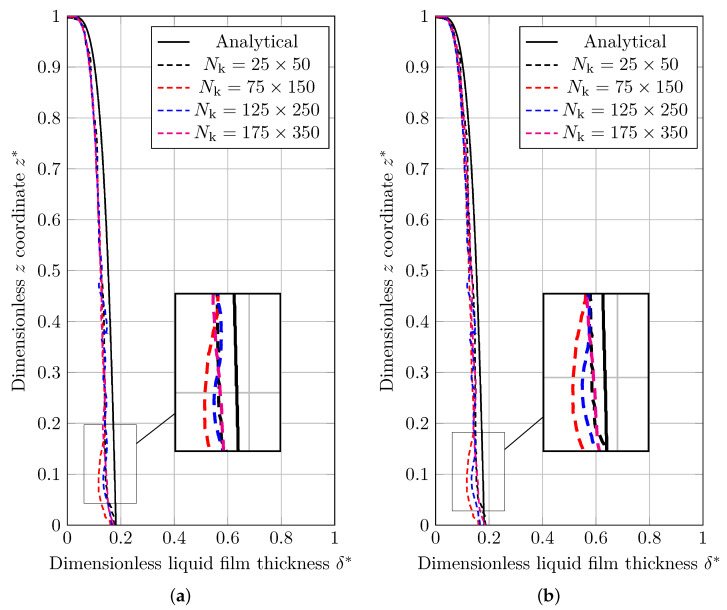
A comparison of the analytical solution and numerical results for the normalized thickness of condensed film on a vertical wall simulated using (**a**) MULES interface description method, and (**b**) isoAdvection interface description method.

The dimensionless results for the thickness of condensed liquid film using the MULES and isoAdvection methods are given in Figure 10a,b, respectively. As demonstrated, simulation results for MULES and isoAdvection methods show some distance with analytical results. As the grid is refined, these distances get smaller, and the results converge to the analytical results.

**Figure 11:**
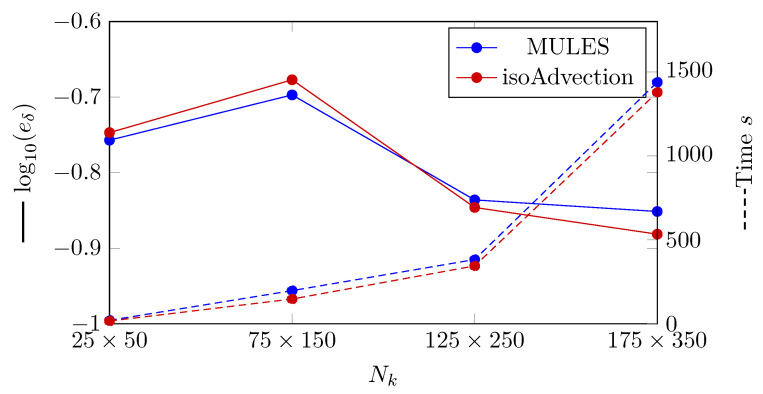
(Solid lines) Logarithmic presentation of the error in which $e_{\delta }$ is the L2 norm of error calculated by Equation (26). (Dashed lines) Computation time of MULES and isoAdvection methods at different grid sizes to solve the vertical film condensation case.

The L2 error is computed and reported in Figure 11 to compare the MULES and isoAdvection techniques in the 2D vertical film condensation benchmark scenario. In this scenario, the following equation defines the L2 error for the thickness of the condensed liquid film as

(28) $$e_{\delta }=\sqrt{\frac{\sum_{i=1}^{N_{k}}{\left(\frac{\delta_{\mathrm{num}},i-\delta_{\mathrm{ana}},i}{\delta_{\mathrm{ana}},i}\right)}^{2}}{N_{k}}},$$

where $N_{k}$ denotes the number of nodes in the *y* direction. As it can be seen in Figure 11, the error produced by isoAdvection is close to MULES. The convergence rate (Equation (18)) between the finest grid structures ($N_{k}=\left[125,175\right]$) for both methods is close to each other and equal to 0.03 for MULES and isoAdvection, respectively. In addition, by focusing on dashed lines in Figure 11, the computational time study can be seen. The isoAdvection method delivers faster computation compared to MULES.

### 3.4. 2D Film Boiling

**Figure 12:**
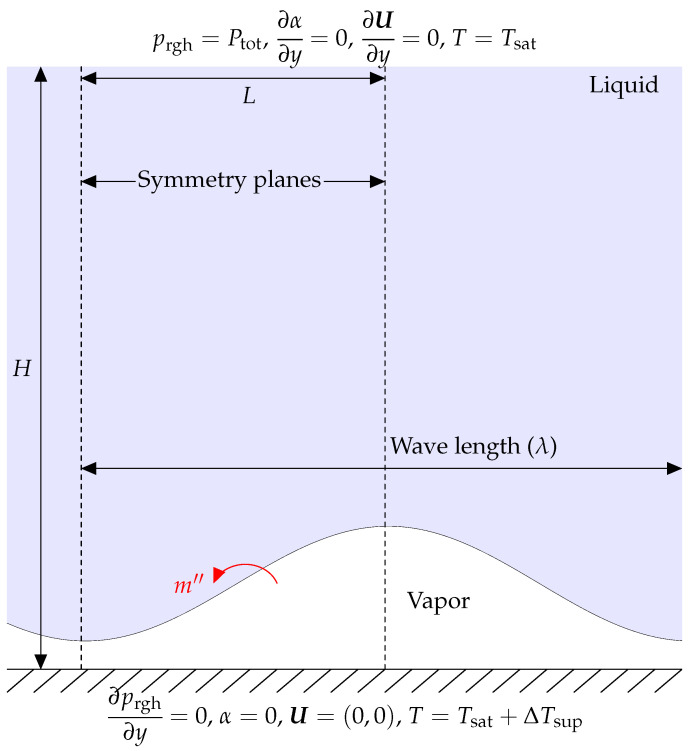
The schematic for the geometry and its boundary conditions in the 2D film boiling case.

Here, a 2D film boiling problem is studied as the study case. As seen in Figure 12, a thin layer of vapor in the shape of a sinusoidal wave exists between the surface and the liquid. The liquid is in the saturation temperature ($T_{\mathrm{sat}}=646$ K), and the surface temperature is higher than the saturation temperature ($\Delta T_{\sup}=5$ K). The liquid–vapor interface is where the phase change occurs, and the resulting vapor then escapes as bubbles from the vapor film layer. The domain is a rectangle with dimensions of L=λ/2 and H=λ. λ is the characteristic length in this case, which is defined as

(29) $$\lambda =\sqrt{\frac{\sigma }{(\rho_{l}-\rho_{v})g}}.$$

The numerical domain has horizontal symmetry, as seen in Figure 12. As a consequence, just a subset of the domain is replicated. A symmetrical boundary condition is stated to exist for vertical boundaries. The pressure outlet condition is applied to the top border, while the vertical gradient of all other variables is zero.

The following equation is used to calculate the initial thickness of the vapor film,

(30) $$\delta =\frac{\lambda_{0}}{64}\left(4+\cos\left(\frac{2\pi x}{\lambda_{0}}\right)\right),$$

where $\lambda_{0}$ is the critical wavelength of the Taylor equation, as determined by

(31) $$\lambda_{0}=2\pi \sqrt{\frac{3\sigma }{(\rho_{l}-\rho_{v})g}}.$$

The Nusselt number is computed as follows:

(32) $$\mathrm{Nu}=\frac{\int_{0}^{L}\left(\frac{\lambda }{\Delta T}\frac{\partial T}{\partial y}{|}_{y=0}\right)\;dx}{L}.$$

Several empirical correlations are used to predict the outcomes of 2D film boiling cases. The experimental correlation provided by [60] is used here. The Nusselt number for 2D film boiling is defined by this correlation as

(33) $$\mathrm{Nu}=0.425\left[\frac{\rho_{v}\left(\rho_{l}-\rho_{v}\right)g\;\left(h_{v}-h_{l}\right)}{k_{v}\mu_{v}\Delta T}\right]$$

To investigate this benchmark situation, four grid structures of $N_{k}$ = [150×300, 175×350, 200×400, 225×450] are selected. Table 2 shows the material parameters utilized to solve the 2D film boiling benchmark scenario. To get dimensionless values, the coordinates *x* and *y* are normalized by scaling with λ (Equation (29)) and *t* is normalized by scaling with $t_{\sigma }$ (Equation (22)).

**Figure 13:**
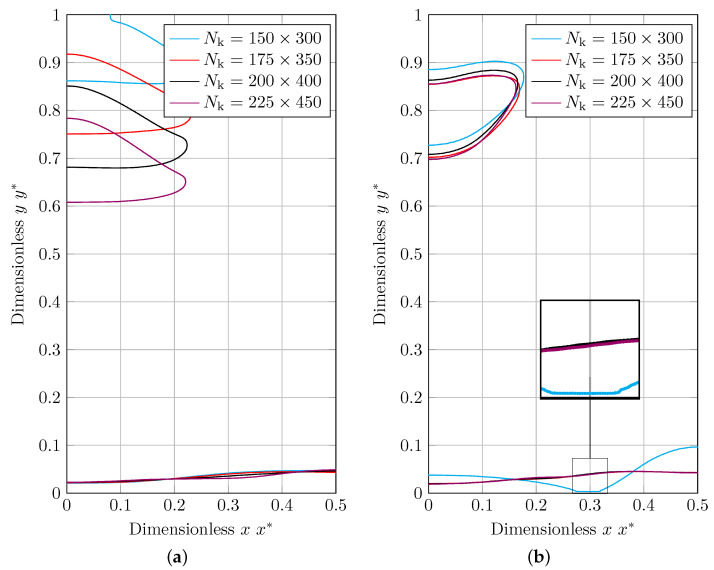
Bubble form in the 2D film boiling benchmark scenario at a certain height for various grid configurations of $N_{k}=\left[150\times 300,175\times 350,200\times 400,225\times 450\right]$, simulated using (**a**) MULES interface description method, and (**b**) isoAdvection interface description method.

In Figure 13, at a specific height, the first separated bubble form is compared for MULES and isoAdvection. These two methods predict approximately the same bubble size but different bubble shapes and bottom curvatures.The detachment and rising bubbles result in an upward flow and the formation of a low-pressure region in their wake. It creates vortices at the bubbles’ sharp edges and has an effect on the bottom curvature. The generated upward flows differs between the MULES and isoAdvection techniques owing to the presence of parasitic currents at varying levels. It describes the various bubble forms in the MULES and isoAdvection techniques.

Another distinction between two approaches is the convergence. This difference becomes considerable as the number of grids increases. While the grid structure is getting finer, the bubble frames are becoming rather closer to each other in the isoAdvection graph. In contrast, there is no such convergence trend seen in MULES, showing the better convergence performance of isoAdvection.

Another result that we are concerned with in Figure 13b is that the isoAdvection predicted film in the coarsest grid structure ($N_{k}=150\times 300$). In the zoomed view of this graph, it can be seen that the interface is attached to the bottom wall. This kind of behavior does not fit in this benchmark case and has not been observed with the MULES and different grid densities shown in Figure 13b. It shows that the isoAdvection method is not working correctly if the grid structure is coarse and the interface approaches the wall. In the following results for the Nusselt number, isoAdvection results for grid structure of $N_{k}=150\times 300$ will thus not be discussed.

**Figure 14:**
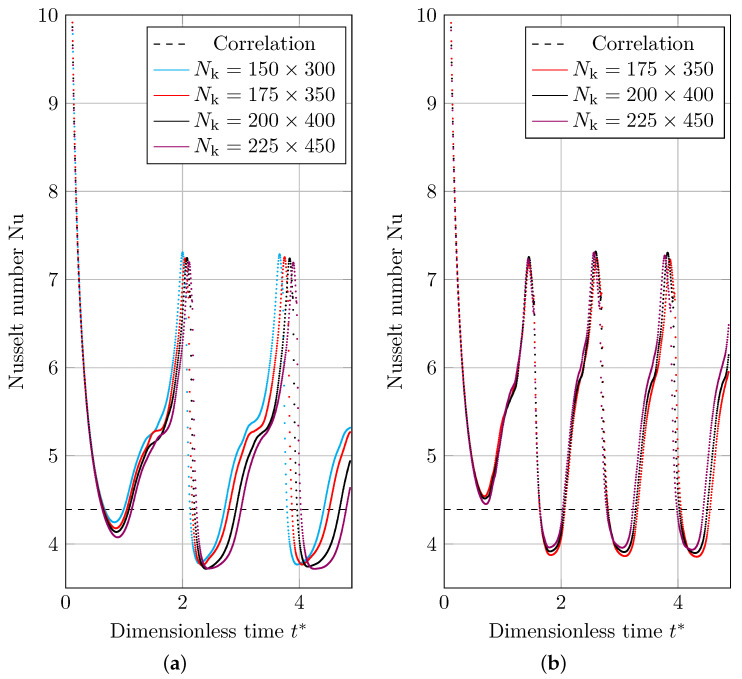
Space-averaged Nusselt number results for 2D film boiling benchmark case (correlation in the legends shows Nusselt number results obtained by Berenson correlation), simulated using (**a**) MULES interface description method, and (**b**) isoAdvection interface description method.

Figure 14a,b illustrate the space-averaged Nusselt number through dimensionless time. The Nusselt number is strongly influenced by the thickness of the film. Heat flow is enhanced when the vapor layer is thin; heat flux is lowered when the film is thick. As the vapor rushes to fill the bubble and the leftover layer thins, the average heat flow and Nusselt number rise. The vapor, on the other hand, returns to the superheated wall after detachment, resulting in a lower Nusselt number due to greater film thickness.

**Figure 15:**
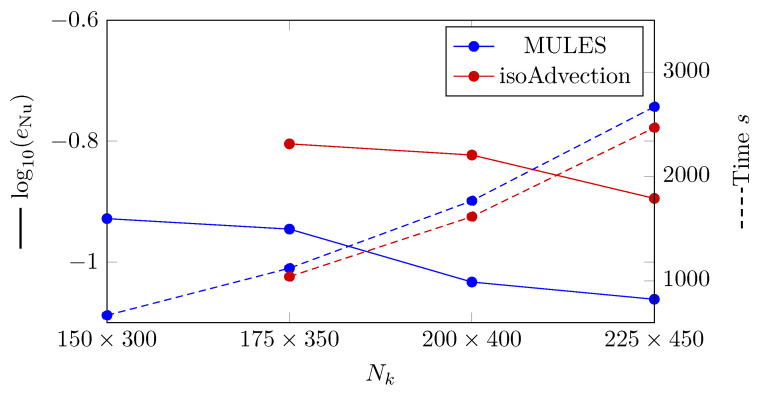
(Solid lines) Logarithmic presentation of the error in which $e_{\mathrm{Nu}}$ is the L2 norm of error in calculating the mean Nusselt number compared to the Berenson correlation calculated by Equation (26). (Dashed lines) Computation time of MULES and isoAdvection methods at different grid sizes to solve the 2D film boiling case.

For MULES and isoAdvection in various analyzed grid configurations, the error between the time-averaged Nusselt number and the mean Nusselt number computed by Berenson correlation (Equation (33)) is displayed (Figure 15). In this situation, the equation that defines the error for the time-averaged Nusselt number ($e_{\mathrm{Nu}}$) is presented as

(34) $$e_{\mathrm{Nu}}=\frac{\sum_{i=1}^{N_{\delta t}}\frac{{\mathrm{Nu}}_{\mathrm{num}},i-{\mathrm{Nu}}_{\mathrm{cor}}}{{\mathrm{Nu}}_{\mathrm{cor}}}}{N_{\delta t}},$$

where $N_{\Delta t}$ represents the number of time steps, ${\mathrm{Nu}}_{\mathrm{num}},i$ represents the Nusselt number in the number i time step, and ${\mathrm{Nu}}_{\mathrm{cor}}$ represents the Nusselt number derived using Berenson correlation. As seen in Figure 15, the error created by isoAdvection is greater than MULES. The convergence rate (Equation (18)) between the finest grid topologies ($N_{k}=\left[200\times 400,225\times 450\right]$) is 0.55 and 1.2 for MULES and isoAdvection, respectively, indicating that the isoAdvection approach has superior convergence.

We compared the calculation time for a bubble detachment period to examine the computational time. The time between the separation of two successive bubbles from the vapor layer is known as the bubble detachment period. The computational time research is shown in Figure 11 by dashed lines. As can be shown, the isoAdvection approach computes quicker than MULES for the detachment of a single bubble.

## 4. Conclusions

**Figure 16:**
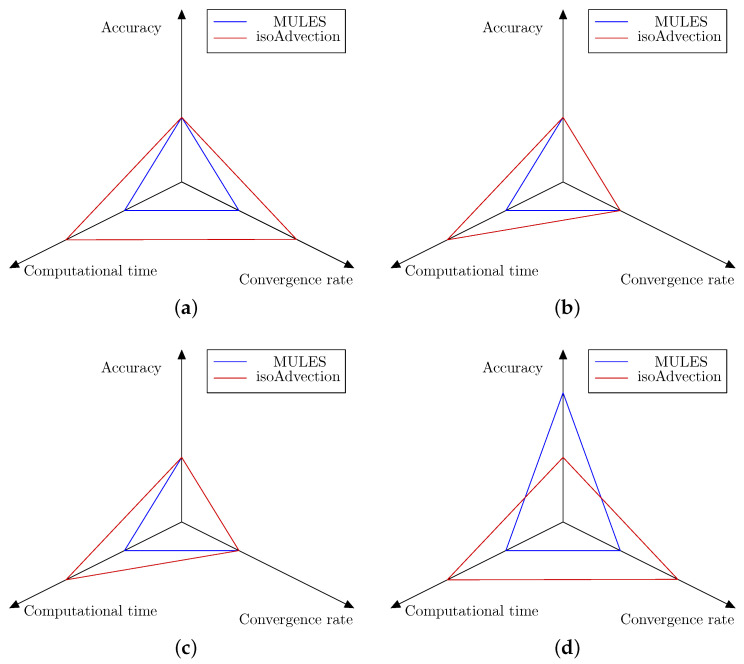
Graphical presentation of MULES and isoAdvection methods performance in accuracy, computational time and convergence rate areas for four thermal phase change benchmark cases. As shown, each axis presents a study area and has two values. If any of the methods has the superior performance in that area it will get the second value, and the first value goes for the inferior performance. (**a**) Stefan problem. (**b**) Horizontal film condensation. (**c**) 2D vertical film condensation. (**d**) 2D film boiling.

The default phase change solvers in OpenFOAM, as well as numerical studies on flow boiling of nanofluids, use VOF-MULES to capture interfaces, resulting in smeared interfaces, spurious flows, and erroneous conclusions. VOF-isoAdvection is a relatively recent technique for determining the interface position. Quantitative validation of the isoAdvection strategy against MULES was performed. Four case studies were compared using their analytical and experimental correlations. The first test case is the Stefan issue (a single-dimensional boiling problem); the second is horizontal film condensation (a single-dimensional condensation problem); the third is condensation on a vertical wall (a 2D condensation problem); and the fourth is film boiling (a 2D boiling problem). These test cases were chosen to illustrate various conditions of thermal phase shift. To conclude the benchmark analysis on these examples, Figure 16 compares the accuracy, computation time, and convergence rate of the isoAdvection and MULES approaches. As stated in the article and concluded in Figure 16, with the exception of the 2D film boiling scenario, where the MULES approach produces more accurate simulation results, the isoAdvection and MULES methods provide comparable results in terms of accuracy and convergence rates. isoAdvection achieves this similar error and convergence performance while always performing better in terms of computation time and simulation speed. Utilizing isoAdvection in applications including condensation, pool boiling, and flow boiling in microchannels could be regarded as a future endeavor.

## Figures and Tables

**Table 1:** Fluid characteristics utilized in the Stefan problem and horizontal film condensation benchmarks.

	Dimension	Base Fluid	Nanoparticles	Vapor
Thermal conductivity, *k*	$W\;m^{2}\;K^{-1}$	0.648	36	0.03643
Density, ρ	$\mathrm{kg}\;m^{-3}$	645	3600	5.1450
Viscosity, μ	Pa s	$1.48\times 10^{-4}$		$1.502\times 10^{-5}$
Specific heat capacity $c_{p}$	$\mathrm{kJ}\;{\mathrm{kg}}^{-1}\;K^{-1}$	2.794	0.765	2.687
Latent heat, *h*	$\mathrm{kJ}\;{\mathrm{kg}}^{-1}$	762.52		2777.1
Surface tension, σ	$N\;m^{-1}$	0.045417		

**Table 2:** Fluid characteristics employed to address two benchmark cases: film condensation on a vertical wall and 2D film boiling.

	Dimension	Base Fluid	Nanoparticles	Vapor
Thermal conductivity, λ	$W\;m^{2}\;K^{-1}$	0.531	36	0.538
Density, ρ	$\mathrm{kg}\;m^{-3}$	370.4	3600	242.7
Viscosity, μ	Pa s	$4.53\times 10^{-5}$		$3.23\times 10^{-6}$
Specific heat capacity $C_{p}$	$\mathrm{kJ}\;{\mathrm{kg}}^{-1}\;K^{-1}$	239	0.765	352
Latent heat, *h*	$\mathrm{kJ}\;{\mathrm{kg}}^{-1}$	1963.5		2240
Surface tension, σ	$N\;m^{-1}$	$7.55\times 10^{-5}$		

**Table 3:** Geometric characteristics utilized in the vertical condensation benchmark.

*L*	Domain length	0.5L
*H*	Domain height	3L
δ(0)	Initial condensed film thickness	0.01L

## Data Availability

Data presented in this article are available at request from the corresponding author.
